# Optimization of the decolorization conditions of Rose Bengal by using *Aspergillus niger* TF05 and a decolorization mechanism

**DOI:** 10.1099/mic.0.001128

**Published:** 2022-01-11

**Authors:** Minghui Zhou, Yan Zhang, Yajun Chen, Fangyan Zhang, Daihu Yang

**Affiliations:** ^1^​ School of Life Sciences, Hefei Normal University, Hefei, Anhui 230601, PR China

**Keywords:** *Aspergillus niger*, Rose Bengal, decolorization, optimization, mechanism

## Abstract

*Aspergillus niger* TF05 was applied to decolorize Rose Bengal dye. The effects of carbon source, nitrogen source, metal ion and spore concentration on Rose Bengal treatment with *A. niger* TF05 were studied. A Plackett–Burman design (PBD) and a uniform design (UD) were used to optimize the decolorization conditions of *A. niger* TF05 and enhance its decolorization effect. The mechanism of Rose Bengal decolorization by *A. niger* TF05 was examined by analysing degradation products via UV–visible light spectroscopy, IR spectroscopy and GC-MS. The best decolorization effect was achieved in the single factor test with glucose and ammonium chloride as carbon and nitrogen sources, respectively. Mg^2+^ was an essential ion that could improve the mould ball state and adsorption efficiency if the spore concentration was maintained at 10^6^ spores ml^–1^. The optimal decolorization conditions obtained using the PBD and UD methods were 11.5 g l^−1^ glucose, 6.5 g l^−1^ ammonium chloride, 0.4 g l^−1^ magnesium sulphate, pH 5.8, 28 °C, 140 r.p.m. rotational speed, 0.18 g l^−1^ dye concentration, 0.5 ml of inocula and 120 h decolorization time. Under these conditions, the maximum decolorization rate was 106%. Spectral analysis suggested that the absorption peak of the product changed clearly after decolorization; GC-MS analysis revealed that the intermediate product tetrachlorophthalic anhydride formed after decolorization. The combined use of the PBD and UD methods can optimize multi-factor experiments. *A. niger* TF05 decolorized Rose Bengal during intracellular enzymatic degradation after adsorption.

## Introduction

Dyes are usually soluble in water and are widely used in many industries, such as textile, paper, cosmetics, rubber, plastic, leather and food industries, but they cause numerous environmental problems [1, 2]. Their chemical structures are complex and extremely stable; they are characterized by light, acid and alkali resistance and have other properties [3]. The organic compounds structured in most dyes are hard to degrade and have teratogenic, carcinogenic, mutagenic and other effects [4–6]. They can be transferred to the food chain and thus are threatening human health [7]. As such, wastewater produced in the production and use of dyestuffs must be properly treated.

Physical, chemical and biological methods are mainly used for the decolorization and treatment of dye wastewater [8]. Physicochemical methods are usually applied at the expense of expensive chemicals and equipment; furthermore, they produce a large amount of sludge and consume a high amount of energy [9–11]. By contrast, biological methods are characterized by easy operation, high efficiency, low cost and zero secondary pollution [12–14]. As such, obtaining efficient dye-degrading strains is key to biological treatments.

Previous studies on dye-degrading strains have mainly focused on white rot fungi [15, 16], yeasts and bacteria [10, 17]; dye decolorization by moulds has also been reported [18, 19]. For example, *Aspergillus* [20, 21] can decolorize dyes. Although many studies have been performed on azo, anthraquinone and triphenylmethane dyes, few studies have explored a wide variety of heterocyclic dyes. In particular, heterocyclic water-soluble dyes, such as Rose Bengal and Congo red [22, 23], are difficult to treat. Rose Bengal, also known as tiger red, is a fluorescein dye containing a polyphenylene ring and an oxanthene structure, which is easily soluble in water. It elicits a certain inhibitory effect on bacteria and restricts the growth of mould colonies [24]. It is also used to study the nature of nucleotide binding sites in some enzymes [25, 26] and applied to photoinactivate and identify amino acid residues at the binding sites of some enzymes [27, 28]. This dye is also difficult to degrade, thereby posing a risk to the water environment.

This study aimed to obtain the optimal decolorizing conditions and degradation mechanism of Rose Bengal by analysing the decolorizing factors and decolorization products with *A. niger* TF05 isolated from dye waste. Meanwhile this study also attempted to expand the microbial resources for the biological treatment of oxanthene dye and provide a basis for improving the degradation efficiency and practical application of *A. niger* in oxanthracene dye treatment.

## Methods

### Strain sources and dye

*A. niger* strain TF05 was isolated from dye waste solution by the Microbiology Laboratory of Hefei Normal University, sequenced and identified by Shenggong Bioengineering (Fig S1 and S2, available in the online version of this article).

Rose Bengal (Table S1) was obtained from the National Pharmaceutical Group Chemical Reagent. Its purity was >98% and was used without further purification.

### Basic medium

Basic medium consisted of glucose (12.0 g), peptone (4.5 g), KH_2_PO_4_ (1.2 g), MgSO_4_·7H_2_O (0.6 g), agar (18–25 g) and distilled water (1000 ml) with a natural pH. The medium was sterilized for 30 min under 0.1 MPa.

### Effect of spore suspension

The isolated and preserved *A. niger* TF05 was activated, inoculated into basic medium and cultured on a tube slant at 28 °C for 72 h. The slant was covered with black spores, washed with sterile distilled water to prepare a spore suspension and adjusted to 10^6^ spores ml^−1^. The spore suspensions of different volumes (0.1, 0.2, 0.4, 0.75, 1.7, 3.0, 5.5 ml) were added to the flasks (250 ml) containing 100 ml of 0.05% Rose Bengal basic medium and incubated at 28 °C and 120 r.p.m. for 72 h. Each volume was performed in triplicate, and 0.05% Rose Bengal basic medium without spore suspension was used as a blank control. Absorbance was determined at 546 nm, and the decolorization rate was calculated.

### Single factor test

Different kinds of carbon and nitrogen sources and metal ions were selected for single-factor decolorization conditions, and their contents were the same as those in the basic medium (Table S2). In this procedure, 100 ml of 0.05 % Rose Bengal was selected in each combination medium with three parallel sample sets, and the corresponding combinations without spore suspension were taken as a blank control. A volume of spore suspension with 10^6^ spores ml^−1^ was inoculated and incubated at 28 °C and 120 r.p.m. for 72 h. The supernatant was obtained through centrifugation at 3000 r.p.m. for 10 min. The OD of the supernatant before (blank control) and after decolorization (parallel samples) was detected at 546 nm, and the decolorization rate was calculated.

### Plackett–Burman design and uniform design [29, 30]

The factors of carbon and nitrogen sources, metal ion, pH, temperature, rotational speed, decolorization time, dye concentration and inoculation amount of the spore suspension were chosen and optimized on the basis of the single factor test to find the overall optimal conditions of decolorization. The different level combinations of nine factors were set in parallel three times, and the corresponding combinations without spore suspension were taken as a blank control. The decolorization rate was used as the response value. A two-level test was conducted with the Plackett–Burman design (PBD) method to screen the significant factors affecting the decolorization rate for later optimization.

The significant factors from the PBD test were optimized with the uniform design (UD) method to determine the optimal decolorization conditions.

### Data processing and statistical analysis

The decolorization rate (%) was calculated as follows:

$$D=\frac{Initial\;absorbance-Observed\;absorbance}{Initial\;absorbance}\times \%$$

The PBD and UD methods were respectively performed using the Minitab package (version 16) and Uniform Design (version 5.0) to screen significant factors *at P*<0.05. A multivariate equation was generated and solved with Matlab (version R2012a).

### Determination of the decolorization mechanism of Rose Bengal by *A. niger* TF05

In this procedure, 1 ml of a spore suspension of 10^6^ spores ml^−1^ was inoculated in 100 ml of 0.05% Rose Bengal basic medium and cultured at 28 °C and 120 r.p.m. for 120 h. At 72 h of culture, observation was carried out every 24 h until the 120 h time point was reached. One sample without spore suspension was set as the blank control, and three parallel samples with spore suspension were set. The cultures of the blank control and parallel samples were collected at 120 h and centrifuged at 10000 r.p.m. for 10 min, and the supernatants were scanned with a UV-vis spectrophotometer (200–800 nm); the supernatants were dried to obtain a powder form, and mixed with KBr powder at a ratio of 1 : 100, scanning analysis was conducted with an IR spectrometer in the range of 400–4000 cm^−1^, and changes in the UV-vis and IR spectra were compared between before (blank control) and after degradation (parallel samples).

GC-MS technology was performed to analyse the biodegradation products of *A. niger* TF05. Powder from blank control and parallel samples was dissolved in chromatographic ethanol for GC-MS analysis, the blank control was scanned once, and three parallel samples were scanned once each, and the mass spectrum changes between before (blank control) and after degradation (parallel samples) were compared. The decolorized products were examined with Trace GC Ultra and ISQ Ⅱ MS. The GC-MS column was a TG-35 ms quartz capillary column, and the column temperature was set from 100 to 300 °C (10 °C min^−1^). The flow rate of the carrier gas (helium) was 1 ml min^−1^. The syringe and detector temperatures were both set at 300 °C. The decolorized products were evaluated by MS under the conditions of an EI mode and 70 eV.

## Results

### Effect of spore suspension and metal ions on decolorization of *A. niger* TF05

**Fig. 1.**
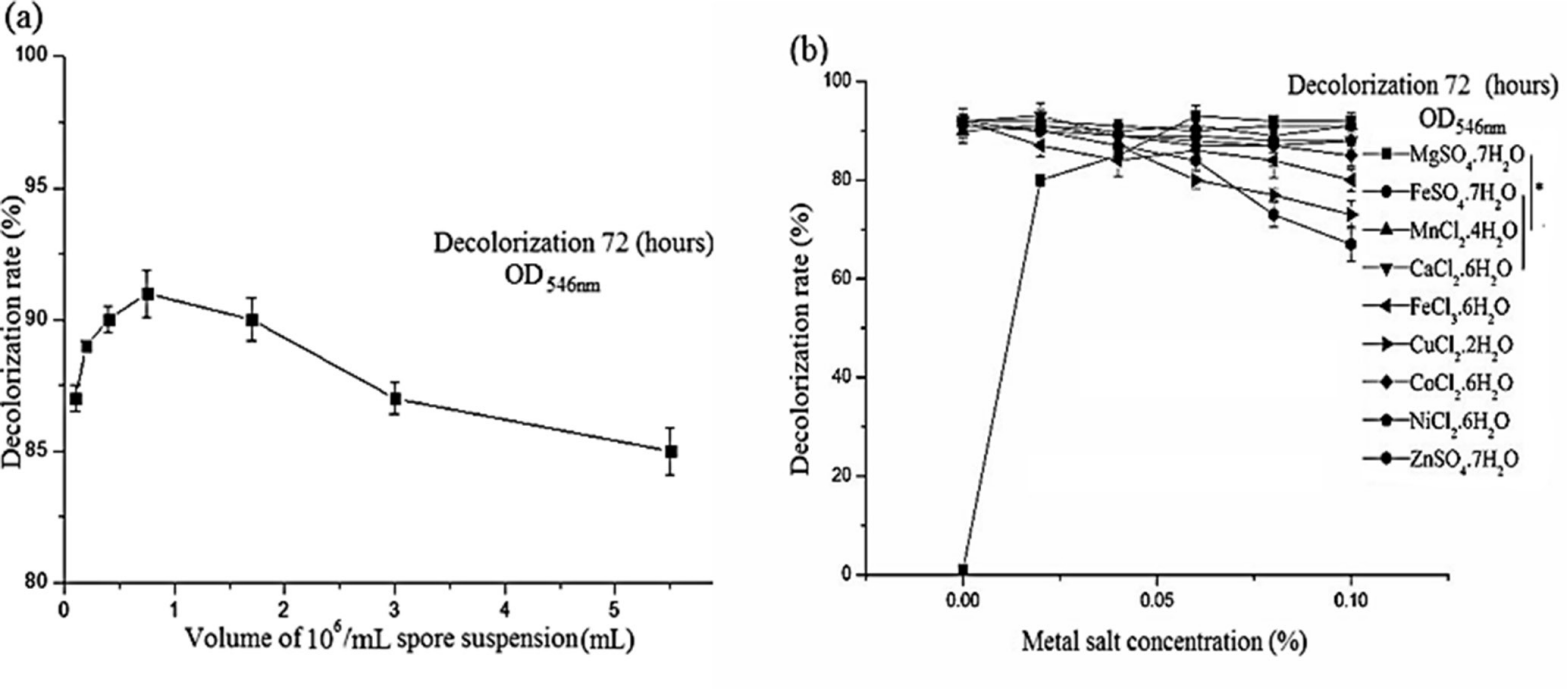
Effect of the volume of spore suspension and metal ions on decolonization of *A. niger* TF05. Spore suspensions of 10^6^ spores ml^−1^ of different volumes and nine metal ions of different masses were added to 100 ml of 0.05 % Rose Bengal basic medium, respectively, and incubated for 72 h. Absorbance of the supernatant was determined at 546 nm and the decolorization rate was calculated. (**a**) Effect of volume of a 10^6^ spores ml^–1^ spore suspension on decolonization. (**b**) Effect of metal ions on decolorization. The data represent the average and sd (shown by error bars) of three biological replicates. Statistical analyses were performed using a one-way ANOVA and significance was determined as *P<0.05.

Decolouring for 72 h, with 0.5–1 ml of a spore suspension of 10^6^ spores ml^–1^, the decolorization rates all exceeded 90%, and had the best decolorization effect (Fig. 1a). The mould balls formed at the low spore suspension concentration had a larger diameter and a looser structure than those at the high spore concentration.

In Fig. 1(b), Fe^2+^, Mn^2+^, Ca^2+^, Co^2+^ and Ni^2+^ had no obvious effect on Rose Bengal decolorization by *A. niger* TF05, and the decolorization rate remained stable as concentration increased. For decolorization, Mg^2+^ was significantly different from Fe^2+^, Mn^2+^ and Ca^2+^. Mg^2+^ plays an crucial role in supporting the growth of *A.niger* TF05. Without Mg^2+^, mould balls cannot form, and consequently Rose Bengal cannot be decolorized. Hence, Mg^2+^ is a key metal ion for Rose Bengal decolorization by *A. niger* TF05. An increase in the concentration of Zn^2+^, Fe^3+^ and Cu^2+^ can gradually slow down the decolorization rate and weaken the decolorization effect.

### Effects of carbon and nitrogen sources on decolorization of *A. niger* TF05

Decolorization is strongly affected by carbon and nitrogen sources (Table 1). The decolorization performance of sugars as a carbon source was better than that of other carbon sources. Glucose, as a carbon source, gave the best decolorization effect with a decolorization rate of 91.59%. Adsorption of the mould ball was better when ammonium salt was used as a nitrogen source than when other nitrogen sources were utilized. Ammonium chloride as a nitrogen source had the best decolorization performance with a decolorization rate of 93.80%. Therefore, glucose and ammonium chloride were chosen as carbon and nitrogen sources for decolorization by *A. niger* TF05.

**Table 1.** Effects of carbon and nitrogen sources on decolorization of *A. niger* TF05

Carbon source	Decolorization rate (%)	Nitrogen source	Decolorization rate (%)
Glucose	91.59±1.11	Peptone	91.97±2.18
Lactose	90.25±2.37	Yeast extract	81.84±2.56
Sucrose	88.05±0.81	Ammonium sulphate	93.09±1.11
Starch	90.59±1.24	Casein	91.78±3.76
Beef paste	55.03±2.48	Ammonium oxalate	90.92±2.89
Sodium citrate	46.24±3.11	Urea	26.74±1.61
Rochelle salt	34.63±1.45	Ammonium chloride	93.80±2.73
Manifold	79.18±2.61	Potassium nitrate	90.71±5.11
Sodium acetate	58.55±4.28	Glycine	80.84±1.78

### Optimization of decolorization conditions

Nine factors were chosen on the basis of the experimental results to determine the optimal conditions, and the decolorization rate was used as a response value. The two-factor PBD test was designed with the Minitab software (Table 2). The Minitab software was used to establish a regression model and perform ANOVA based on the data in Table 2, and the results are shown in Table 3.

**Table 2.** Placket–Burman test design and response value

Test no.				Factor level						
	Glucose A (g l^−1^)	Ammonium chloride B (g l^−1^）	Magnesium sulphate C (g l^−1^)	pH D	Temperature E (°C)	Rotational speed. F (r.p.m.)	Decolorization time G (h)	Dye concentration H (g l^−1^）	Amount of inoculum (ml^−1^ 100 ml)	Decolorization rate (%)
1	10	4	0.6	6	24	100	120	0.22	1.5	56.0±1.27
2	8	6	0.4	6	24	140	120	0.22	0.5	63.4±2.11
3	10	6	0.6	6	28	140	72	0.22	0.5	67.3±1.91
4	8	4	0.6	8	28	100	120	0.22	0.5	51.0±3.17
5	8	4	0.4	8	28	140	72	0.22	1.5	50.0±1.08
6	10	6	0.4	8	24	100	72	0.22	1.5	52.0±2.12
7	9	5	0.5	7	26	120	96	0.2	1.0	90.2±4.25
8	9	5	0.5	7	26	120	96	0.2	1.0	85.4±2.05
9	9	5	0.5	7	26	120	96	0.2	1.0	88.4±4.08
10	10	4	0.6	8	24	140	72	0.18	0.5	52.2±1.58
11	8	6	0.6	6	28	100	72	0.18	1.5	64.1±1.96
12	8	4	0.4	6	24	100	72	0.18	0.5	55.1±3.28
13	8	6	0.6	8	24	140	120	0.18	1.5	55.2±2.86
14	10	6	0.4	8	28	100	120	0.18	0.5	63.3±3.27
15	10	4	0.4	6	28	140	120	0.18	1.5	70.1±2.54

**Table 3.** Analysis of significance of various factors

Standard	Effect	Coefficient	Coefficient standard error	*T*	*P*
Constant		58.308	0.5572	104.65	0.000
A	3.683	1.842	0.5572	3.31	0.030
B	5.150	2.575	0.5572	4.62	0.010
C	−1.350	−0.675	0.5572	−1.21	0.292
D	−8.717	−4.358	0.5572	−7.82	0.001
E	5.317	2.658	0.5572	4.77	0.009
F	2.783	1.392	0.5572	2.50	0.067
G	3.050	1.525	0.5572	2.74	0.052
H	−3.383	−1.692	0.5572	−3.04	0.039
I	−0.817	−0.408	0.5572	−0.73	0.504

Coefficient of determination: *R* ^2^=0.98.

The obtained coefficient of determination (*R* ^2^) was 0.98, indicating that the model explained 98% of the variation in the response. ANOVA of the decolorization rate yielded *P*<0.05, suggesting that the model was significant. Glucose, ammonium chloride, pH, temperature and dye concentration were significant factors affecting the decolorization capability of *A. niger* TF05 (*P*<0.05). Other factors, including rotational speed (140 r.p.m.), decolorization time (120 h), magnesium sulphate concentration (0.4 g l^−1^) and inoculum volume (0.5 ml), were selected on the basis of constant positive and negative effects.

The optimal design for glucose, ammonium chloride, pH, temperature and dye concentration from the PBD test was subjected to the UD method to obtain the optimal values. The UD table and analysis results are shown in Tables 4 and 5, respectively.

**Table 4.** Uniform design and response values

Test no.		Factor level				Decolorization rate (%)		
	Glucose (X_1_) (g l^−1^)	Ammonium chloride (X_2_) (g l^−1^)	pH (X_3_)	Temperature (X_4_) (°C)	Dye concentration (X_5_) (g l^−1^)	Y_1_	Y_2_	Y_3_
1	10.0	6.5	4.0	34	0.18	82	80	72
2	10.5	7.5	6.0	32	0.16	92	94	90
3	11.0	8.5	3.5	30	0.14	73	69	78
4	11.5	9.5	5.5	28	0.12	76	76	81
5	12.0	6.0	3.0	35	0.10	78	75	80
6	12.5	7.0	5.0	33	0.08	96	97	93
7	13.0	8.0	2.5	31	0.06	48	40	54
8	13.5	9.0	4.5	29	0.04	40	42	51

**Table 5.** Significance test of the regression equation system

Independent variable	Coefficient	Standard error of coefficient	*T*	*P*
Constant	−1266.7	165.0	−7.68	0.000
X_1_	197.94	21.82	9.07	0.000
X_2_	41.06	14.41	2.85	0.011
X_3_	35.213	7.938	4.44	0.000
X_1_^2^	−8.6111	0.9275	−9.28	0.000
X_2_^2^	−3.1667	0.9275	−3.41	0.003
X_3_^2^	−3.0556	0.9275	−3.29	0.004

Coefficient of determination: *R^2^* =0.936.

The binary regression model of the test results in Table 4 was established with the Minitab software, and the regression equation was expressed as follows:

$$Y=-1267+198X_{1}+41.1X_{2}+35.2X_{3}-8.61X_{1}^{2}-3.17X_{2}^{2}-3.06X_{3}^{2}\cdot \;\;\;\;\;\;(1)$$

A significance test and analysis of regression model variance of each factor of the test results were carried out (Table 5).

The quadratic model had *P*=<0.001, indicating that the model was significant (Table 5). The model equation derived with UD and Minitab 16 could be adequately used to describe the optimization of decolorization conditions. *R* ^2^ was 0.936, which could explain up to 93.6% of the variability of the response and meet the test requirements. Matlab was used to solve Equation (1) and obtain optimal values of 11.5 g l^−1^, 6.483 g l^−1^ and 5.752 for the amount of glucose, ammonium chloride and pH, respectively. The maximum decolorization rate was 106%. The effects of temperature and dye concentration on the test results were highly coincident with the three other factors, so they were automatically eliminated by the software. According to the effect value of the PBD test, the dye concentration and temperature were 0.18 g l^−1^ and 28 °C respectively. The obtained optimal decolorization conditions using the PBD and UD methods were 11.5 g l^−1^ glucose, 6.5 g l^−1^ ammonium chloride, 0.4 g l^−1^ magnesium sulphate, pH 5.8, 28 °C, 140 r.p.m. rotational speed, 120 h decolorization time, 0.5 ml of inoculum and 0.18 g l^−1^ dye concentration. Under the optimal decolorization conditions, the verification test showed that the decolorization rate was up to 100%, which approximated to the theoretical value.

### Decolorization mechanism of Rose Bengal by *A. niger* TF05

**Fig. 2.**
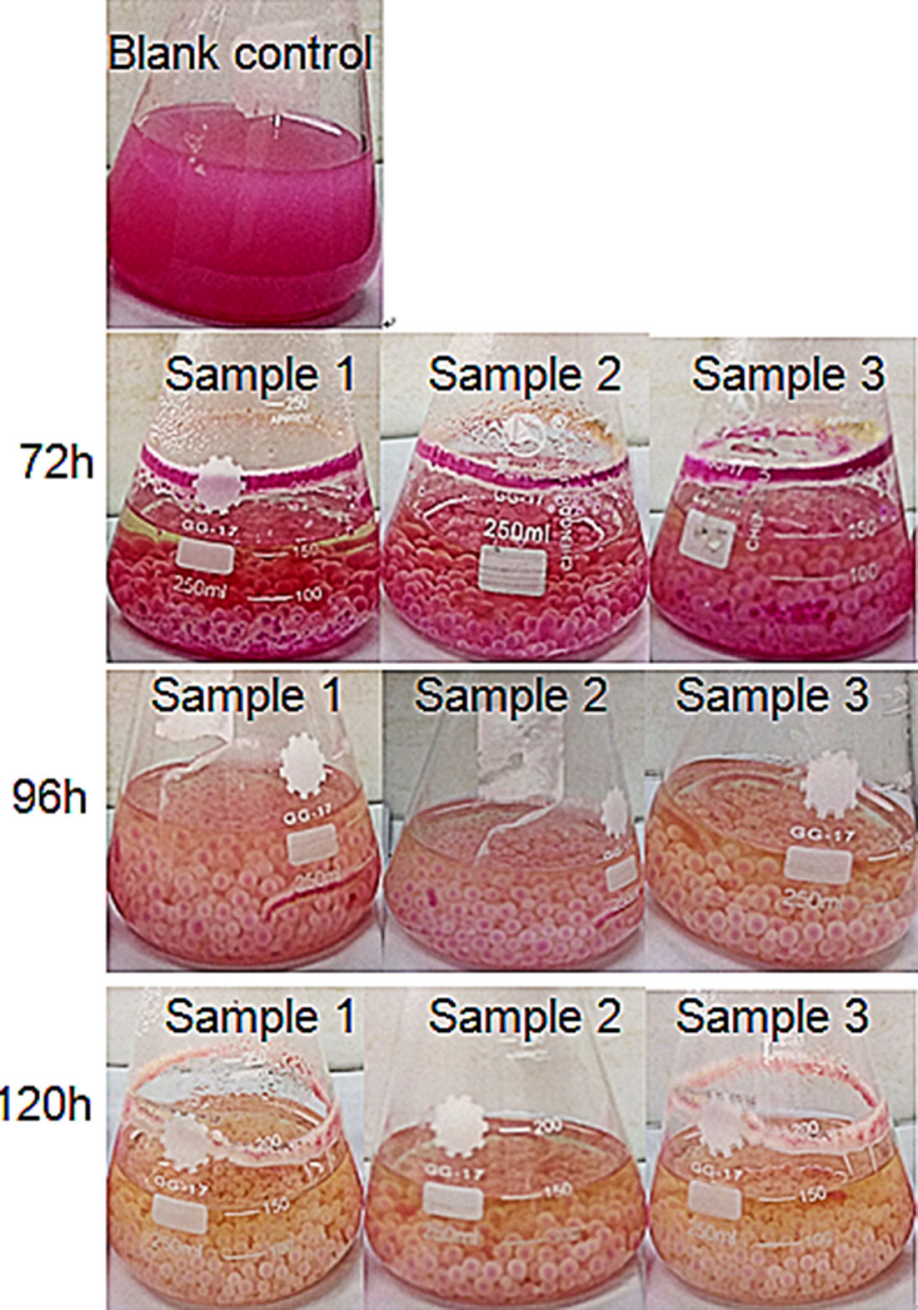
Decolorization process of *A. niger* TF05. A spore suspension (1 ml of 10^6^ spores ml^−1^) was inoculated in 100 ml of 0.05% Rose Bengal basic medium and incubated for 120 h. Three parallel samples and a blank control (without spore suspension) were set up. The decolorization process was observed at 72, 96 and 120 h.

Three parallel samples were decolorized for 72 h, three consecutive observations were made every 24 h, and we recorded the decolorization process (Fig. 2). Fig. 2 shows that *A. niger* TF05 spores formed relatively complete mycelium pellets after 72 h of oscillatory culturing, and the fungus pellets contain dyes. A large amount of Rose Bengal dye was absorbed in the balls, and the colour of the culture media lightened after 96 h. After 120 h, the dye in the balls was completely degraded, and the culture media were completely cleared, indicating that Rose Bengal decolorization by *A. niger* TF05 involved adsorption and degradation.

**Fig. 3.**
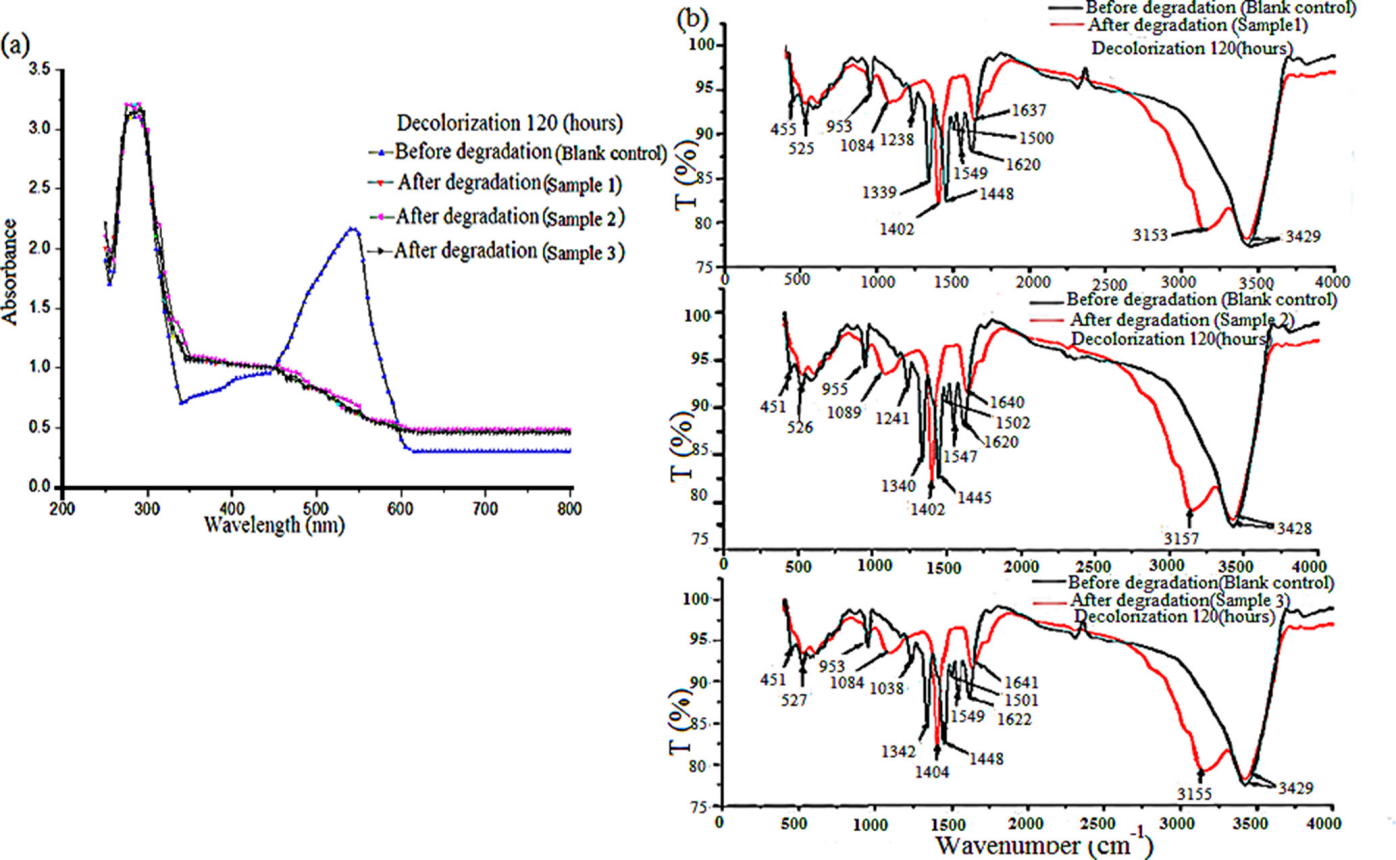
UV–vis and IR absorption spectrum before and after decolorization of Rose Bengal by *A. niger* TF05. After decolorization for 120 h, the culture solution of three parallel samples and a blank control (without spore suspension) were centrifuged to obtain the supernatant. The supernatant was scanned with a UV–vis spectrophotometer (200–800 nm). The remaining supernatant after UV–vis detection was dried into powder and scanning analysis was conducted with an IR spectrometer in the range of 400–4000 cm^−1^. (**a**) UV–vis absorption spectrum before and after decolorization of Rose Bengal by *A. niger* TF05. (**b**) IR absorption spectrum before and after decolorization of Rose Bengal by *A. niger* TF05.

Supernatants of the blank control and three parallel samples were scanned in the range of 200–800 nm with UV–vis light. The absorption spectra of the three samples were generally similar. The absorption spectrum of Rose Bengal changed notably after it was decolorized by *A. niger* TF05 (Fig. 3a), The UV-vis absorption spectra of the three samples were consistent; the fine structure of the benzene ring at 250–300 nm did not change before (blank control) or after (parallel samples) decolorization, and it had an obvious absorption peak, indicating that the benzene ring is still present after degradation; after decolorization, the absorption peak at 546 nm of three parallel samples disappeared completely, without other obvious absorption peaks being formed; 546 nm is the absorption peak of the oxanthracene structure, suggesting that this structure has been damaged. The absorbance increased at 350–450 nm, and new substances might be produced, indicating that the molecular structure of Rose Bengal changed after decolorization. The obvious change of the UV–vis absorption spectrum showed that Rose Bengal decolorization by *A. niger* TF05 was caused by biodegradation. The powder of the blank control and three parallel samples was IR scanned in the range of 500–4000 cm^−1^ to study the changes in the molecular structure of Rose Bengal before and after decolorization (Fig. 3b).

In Fig. 3(b), the IR spectrum before degradation (blank control) showed that the stretching vibration absorption of C–O was detected at 1238 and 1340 cm^−1^, and four sharp absorption peaks with different intensities were found at 1448, 1500, 1548 and 1620 cm^−1^, which are important characteristics of aromatic oxanthracene compounds [31]. After degradation, the IR spectrum of the three parallel samples were generally similar, with only the wavelength corresponding to the characteristic peaks having changed slightly. The characteristic peaks of aromatic compounds decreased notably after degradation (parallel samples), indicating that open chain breakage might have occurred. The weak peaks between 500 and 953 cm^−1^ before and after degradation were the absorption peaks of C–Cl and C–I, and the peak shape changed slightly, suggesting that C–Cl and C–I have not been damaged, but the content is reduced [31]. A wide peak at 3428 cm^−1^ before and after degradation with different intensities might be attributed to the moisture of the sample that was not treated completely, consequently showing a water peak of –OH. After degradation, clear absorption peaks appeared at 1084, 1402, 1640 and 3155 cm^−1^, followed by C–O, C–N, C=O and N–H stretching vibration peaks [31]; the results also showed that the shape of the peak and the molecular structure of the Rose Bengal changed notably after degradation, and the molecular structure of the degraded product might contain C–O, C–N, C=O and N–H.

**Fig. 4.**
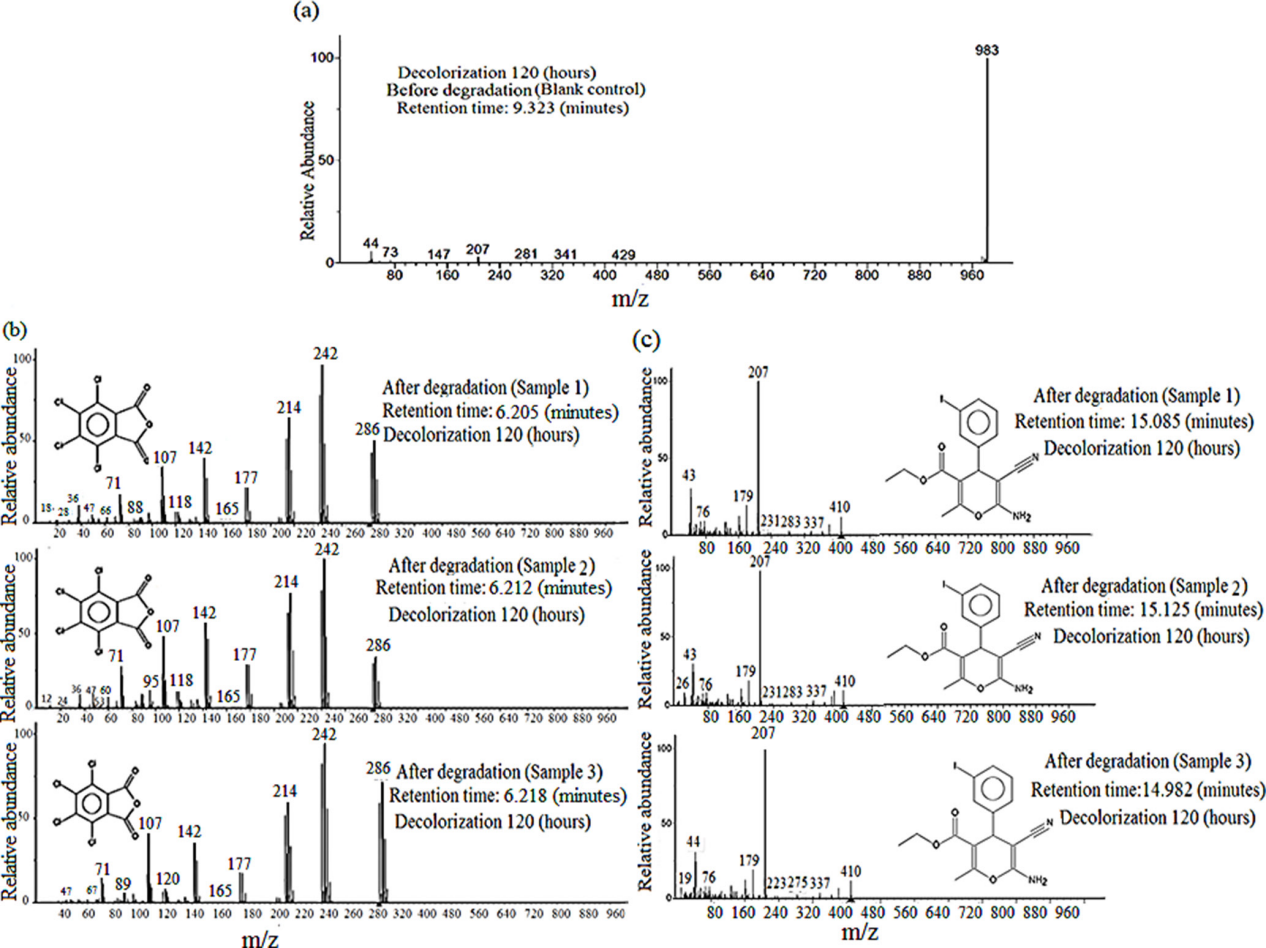
GC-MS pattern before and after Rose Bengal degradation by *A. niger* TF05. Residual powder after IR detection, including three parallel samples and a blank control, dissolved in chromatographic ethanol for GC-MS analysis, and the syringe and detector temperatures were both set at 300 °C. Three parallel samples and a blank control were evaluated through MS under the conditions of an EI mode and 70 eV. (**a**) GC-MS pattern before Rose Bengal degradation (blank control). (**b**) GC-MS pattern of the main degraded products after Rose Bengal degradation (parallel samples) by *A. niger* TF05. (**c**) GC-MS pattern of the minor degraded products after degradation (parallel samples) of Rose Bengal by *A. niger* TF05.

Further GC-MS analysis of the products before degradation (blank control) (Fig. 4a) showed that the main substance was a molecular fragment of Rose Bengal with a molecular weight of 983 and a retention time of 9.323 min; no obvious change in the chemical structure of Rose Bengal was observed. After degradation, the mass spectrum of three parallel samples were generally the same, with only the molecular weights of the ion fragments being slightly different (Fig. 4b, c) . The main component detected after degradation (parallel samples) was tetrachlorophthalic anhydride (molecular weight of 286 and retention time of 6.212±0.0065 min), and its characteristic molecular fragments were 36, 71, 107, 142, 177, 214 and 242 in size (Fig. 4b). Comparison of the detected substance with the standard substance in the GC-MS NIST database revealed that tetrachlorophthalic anhydride shared a high degree (93.5%) of similarity with the standard substance. Therefore, tetrachlorophthalic anhydride was the main product of Rose Bengal degradation. In addition to the main degradation product, 6-amino-5-cyano-4- (3-iodo-phenyl)−2-methyl-4H-pyran-3-carboxylate (molecular weight of 410 and retention time of 15.063±0.0735 min) was detected with a low matching degree (7.67%) in the NIST database, but C–N and N–H were present in the product, and its characteristic molecular fragments were 43, 76, 179 and 207 in size (Fig. 4c). This observation was consistent with the IR detection spectrum because the compositions of the substance and tetrachlorophthalic anhydride were highly similar to that of Rose Bengal dye. Therefore, the C–C bond between polyphenylene and oxanthracene of Rose Bengal is disrupted under the action of degrading enzymes, forming tetrachlorophthalic anhydride and tetraiodoxanthracene; the latter further degrades smaller molecules. However, the specific degradation path and enzymatic action mechanism need to be further studied.

## Discussion

The mycelium of a mould develops and grows rapidly. The mycelial ball formed is characterized by a wide pH tolerance range, a certain mechanical strength, a strong adsorption capacity and the absence of a carrier; as such, it can be directly used in a fixed bed and fluidized bed, and other reactors, to treat dye-containing wastewater via adsorption [32, 33]. *Penicillium*, *Aspergillus* and *Rhizopus* are utilized to treat dye-containing wastewater. However, *Penicillium* has a weak ball-forming ability, and *Rhizopus* can show good decolorization effect only on low-concentration dyes; by comparison, *Aspergillus* has a better decolorization effect on high-concentration dyes [34, 35]. In our study, the decolorization rate of Rose Bengal dye of 0.18 g l^−1^ by *A. niger* TF05 reached 100% after 120 h, and the removal effect was enhanced.

The PBD two-factor method can quickly and effectively screen the most significant factors and consequently avoid wasting experimental resources because too many factors or some factors are not significant in later optimization experiments [36, 37]. The PBD is generally used with a central composite design (CCD) or a Box–Behnken design (BBD) in the response surface method (RSM) to obtain optimal values for significant factors and response surface graphs [38]. CCD and BBD methods are generally suitable for the optimization of a small number of significant factors. If more than three significant factors are found, the number and the difficulty of experiments will be greatly increased [39]. The UD method involves several experiments in dealing with multifactor and multilevel experimental design problems; with a small number and a more uniform distribution, values can be optimized quickly and effectively [40, 41]. In our experiment, the PBD was combined with the UD, and Minitab and Matlab were used to analyse the results. The significant factors affecting the decolorization conditions of *A. niger* TF05 were obtained with a high efficiency, and the optimal values of the significant factors were accurately calculated. Under the optimal decolorization conditions, the decolorization rate reached 100%. The significant factors screened with the PBD were consistent with the results of the ANOVA of each factor in the UD. Therefore, the combined use of the PBD and UD could play an important role in the screening and optimization of significant factors.

Since the Rose Bengal molecule contains both oxanthracene and triphenylmethane, *A. niger* capable of degrading Rose Bengal may have a wide spectrum of dye degradation. Generally, reactive dyes are highly water-soluble and have a very high chroma after entering the water body without treatment, which affects the water quality [42]. Rose Bengal dye, after being absorbed and degraded by *A. niger*, becomes clear and transparent and has a good decolorization effect. Large organic dye molecules are very difficult to remove directly, whereas *A. niger* can degrade Rose Bengal into smaller molecules, which are easily further treated. The main product of Rose Bengal degradation by *A. niger* TF05 was tetrachlorophthalic anhydride. Phytotoxicity was not obvious (Fig. S3), and its toxicity to the environment should be further studied. However, the degraded product does not dissolve in water and can be precipitated out easily; tetrachlorophthalic anhydride is a type of intermediate used in medicine and dye organic synthesis and can be recycled. Tetrachlorobenzoic anhydride can be converted to tetrachlorobenzoic acid under the action of the oxidase of *Pyrechium crassicum*, which enters the TCA cycle and is eventually mineralized into CO_2_ and H_2_O by microorganisms [43]. Therefore, *A. niger* can co-degrade with other microorganisms to achieve complete degradation.

The analysis of dye intermediates is important in exploring the biodegradation mechanism and degradation pathway of dyes, and bioremediation can be achieved via two mechanisms: biodegradation and biosorption [37]. Many researchers have used white rot fungi for dye degradation [44]. In contrast to the mechanism of dye removal by white rot fungi, the mechanism of dye removal by non-white rot fungi mainly involves adsorption; in some cases, non-white rot fungi are more efficient than white rot fungi [20, 21], In our study, UV–visible light and IR scanning before and after decolorization revealed that the absorption spectrum changed notably, indicating that the absorption peak of the oxygen heteroanthracene structure disappeared at 546 nm. Furthermore, the absorption intensity of the benzene ring decreased, and new bonds formed. GC-MS analysis showed that the main product of Rose Bengal degradation by *A. niger* TF05 was tetrachlorophthalic anhydride.

Experiments showed that neither the single live and dead microspheres nor the supernatant was sufficient to adsorb and degrade Rose Bengal dye. Therefore, enzymatic degradation can occur only when the spore suspension is co-cultured with Rose Bengal. In the process of mycelium growth, the dye molecules are absorbed and wrapped in the mycelium ball. At present, dye-degrading enzymes mainly include azo reductase, lignin peroxidase (LiP), manganese peroxidase (MnP) and lachase (Lac) [45], and the above enzymes are mainly extracellular. Rose Bengal is degraded inside *A. niger* TF05 mould balls, so Rose Bengal degradation enzymes should be intracellular. Currently, there are no reports regarding the enzymatic degradation of the dye, so further study on the degradation enzyme is needed.

## Conclusion

The combination of PBD and UD can quickly obtain the optimal conditions in multi-factor experiments, save experimental resources and reduce experimental costs. The mechanism of Rose Bengal decolorization by *A. niger* TF05 was biodegradation that involved two steps. Rose Bengal was first absorbed into a mould ball and then degraded by related intracellular enzymes into small molecular substances. However, the participation of related degradation enzymes and the formation of specific small molecular degradation products should be further studied.

## Supplementary Data

Supplementary material 1
