# Supplementary material for: Optimization of the decolorization conditions of Rose Bengal by using Aspergillus niger TF05 and a decolorization mechanism
Source: Microbiology (Reading). 2022 Jan 11;168(1):001128. doi: 10.1099/mic.0.001128 (PMC8914245; doi:10.1099/mic.0.001128)
Supplement: Supplementary material 1 [file mic-168-1128-s001.pdf]

Table S1 General characteristics of Rose Bengal

Chemical structure	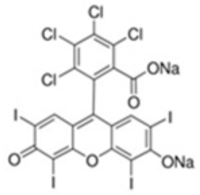
Molecular formula	$C_{20}H_4Cl_4I_4O_5$
Formula weight	1017.64
Color index(C.I.)number	45440
CAS number	632-69-9
λmax	546nm

Table S2. Single factor design of the experiments

Factor	Level
Carbon source	Glucose, Lactose, Sucrose, Starch, Beef paste, Sodium citrate, Potassium sodium Tartrate, Mannitol, Sodium acetate
Nitrogen source	Peptone, Yeast extract, Ammonium sulfate, Casein, Urea,Ammonium chloride, Potassium nitrate, Glycine,
$MgSO_4$•$7H_2O$	0、 0.02、 0.04、0.06、0.08、0.1
$MnCl_2$•$4H_2O$	0、0.01、0.02、0.03、0.04、0.05
$ZnSO_4$•$7H_2O$	0、0.01、0.02、0.03、0.04、0.05
$CaCl_2$•$6H_2O$	0、0.01、0.02、0.03、0.04、0.05
$CoCl_2$•$6H_2O$	0、0.01、0.02、0.03、0.04、0.05
$CuCl_2$•$2H_2O$	0、0.01、0.02、0.03、0.04、0.05
$NiCl_2$•$6H_2O$	0、0.01、0.02、0.03、0.04、0.05
$FeCl_3$•$6H_2O$	0、0.01、0.02、0.03、0.04、0.05
$FeSO_4$•$7H_2O$	0、0.01、0.02、0.03、0.04、0.05

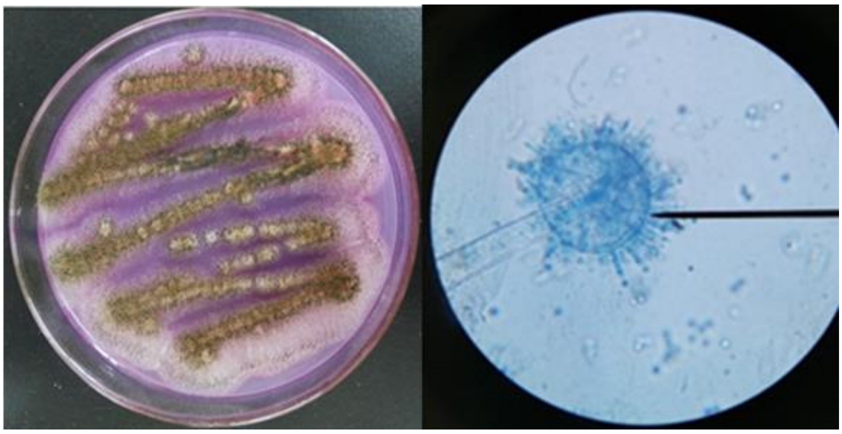

Fig. S1 Colony and spore morphology of strain TF05

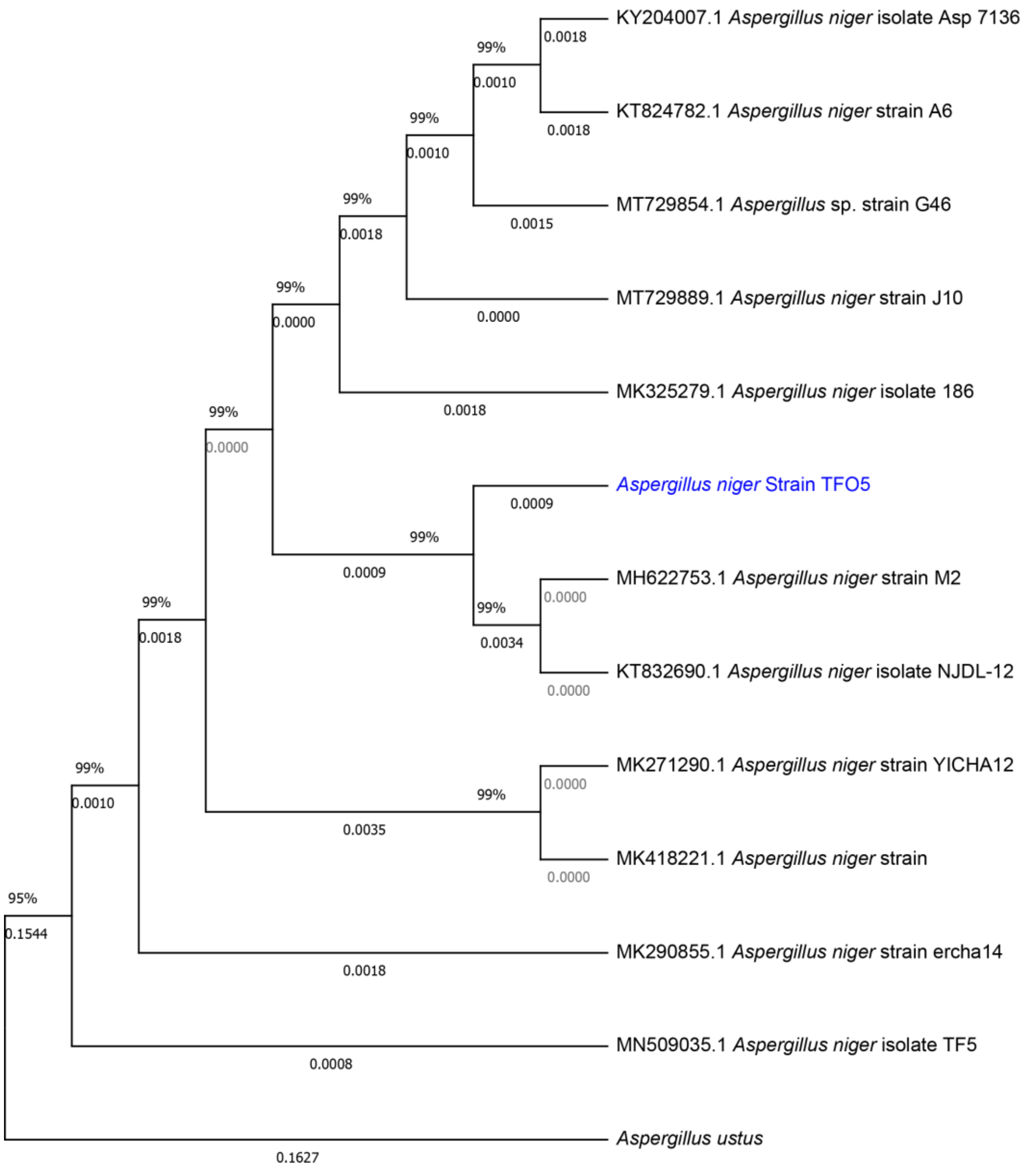

Fig. S2 Gene comparison results of strain TF05

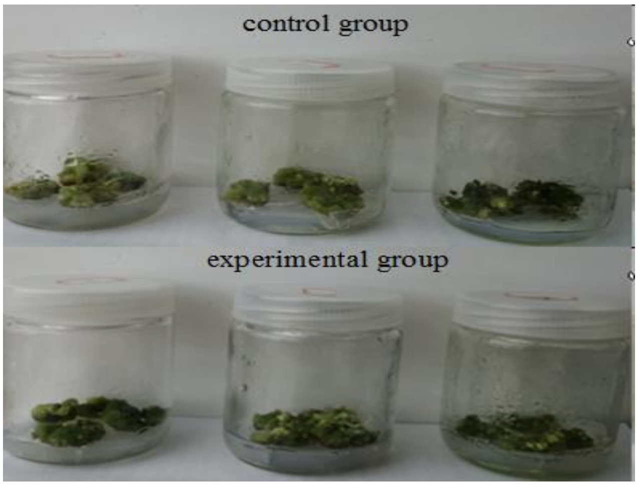

Fig. S3　Phytotoxicity test
